# Supplementary material for: Untargeted Metabolomic Study for Urinary Characterization of Adult Patients with Phenylketonuria
Source: Int J Mol Sci. 2025 Dec 6;26(24):11808. doi: 10.3390/ijms262411808 (PMC12732599; doi:10.3390/ijms262411808)
Supplement: Supplementary file 1 [file ijms-26-11808-s001.zip › ijms-3752443-supplementary.pdf]

SUPPLEMENTARY INFORMATION

Untargeted Metabolomic Study for Urinary Characterization of Adult Patients with Phenylketonuria

Arnau Gonzalez-Rodriguez [1,2], Blanca Barrau-Martinez [1,2], Adriana Pané [3,4,5], Rosa Maria López Galera [4,6,7,8],
Ester Tobias [4,6,8,9], Cristina Montserrat-Carbonell [3,4], Mariona Guitart-Mampel [4,6,8,9], Olga Jáuregui [10],
Regina Roca-Vives [10], Judit Garcia-Villoria [4,6,7,8], Jose Cesar Milisenda [4,6,8,9], Ana Matas-Garcia [4,6,8,9],
Maria de Talló Forga Visa [3,4], Pedro Juan Moreno Lozano [4,6,8,9], Gloria Garrabou [4,6,8,9], Mireia Urpi-Sarda [1,2,11]*,
Rafael Llorach [1,2,11,]*; on behalf of the Consortium PKU.cat†

1 Food and Lifestyle ExposOMics Research Group (FLExOMics-UB), Departament de Nutrició, Ciències de l'Alimentació i Gastronomia, Facultat de Farmàcia i Ciències de l'Alimentació, Campus de l'Alimentació de Torribera, Universitat de Barcelona (UB), 08921 Santa Coloma de Gramenet, Spain. agonzalezrodriguez@ub.edu (A.G.-R.); blancabarrau@ub.edu (B.B.-M.).
2 Institut de Recerca en Nutrició i Seguretat Alimentària (INSA-UB), Campus de l'Alimentació de Torribera, Universitat de Barcelona (UB), 08921 Santa Coloma de Gramenet, Spain.
3 Endocrinology and Nutrition Department, Hospital Clínic of Barcelona, 08036 Barcelona, Spain; pane@clinic.cat (A.P.); cmontse@clinic.cat (C.M.-C.); maforvi@gmail.com (M.d.T.F.V.).
4 Adult Inborn Errors of Metabolism Unit, Hospital Clínic of Barcelona, 08036 Barcelona, Spain; rmlopez@clinic.cat (R.M.L.G.); etobiasb@ub.edu (E.T.); mguitart@recerca.clinic.cat (M.G.-M.); jugarcia@clinic.cat (J.G.-V.); jcmilise@clinic.cat (J.C.M.); anmatas@clinic.cat (A.M.-G.); pjmoreno@clinic.cat (P.J.M.L.); garrabou@clinic.cat (G.G.).
5 Centro de Investigación Biomédica en Red de la Fisiopatología de la Obesidad y Nutrición (CIBEROBN), Instituto de Salud Carlos III, 28029 Madrid, Spain.
6 Fundació Clínic per la Recerca Biomèdica (FCRB), Institut d'Investigacions Biomèdiques August Pi Sunyer (IDIBAPS), 08036 Barcelona, Spain.
7 Division of Inborn Errors of Metabolism-IBC, Biochemistry and Molecular Genetics Department, Hospital Clínic of Barcelona, 08036 Barcelona, Spain.
8 Centro de Investigación Biomédica en Red de Enfermedades Raras (CIBERER), Instituto de Salud Carlos III, 28029 Madrid, Spain.
9 Inherited Metabolic Diseases and Muscle Disorders Research Laboratory, Cellex-IDIBAPS, Faculty of Medicine and Heath Sciences-University of Barcelona, 08036 Barcelona, Spain.
10 Centres Científics i Tecnològics de la Universitat de Barcelona (CCiTUB), 08028 Barcelona, Spain; regiroca@ccit.ub.edu (R.R.-V.).
11 Centro de Investigación Biomédica en Red de Fragilidad y Envejecimiento Saludable (CIBERFES), Instituto de Salud Carlos III, 28029 Madrid, Spain.
* Correspondence: murpi@ub.edu (M.U.-S.); rafallorach@ub.edu (R.L.)
† Collaborators of the Consortium is provided in the Acknowledgments section.

Materials and Methods

Plasma sample preparation

Plasma samples (100 μL) were processed following previously established protocols (1,2), using acidic solvent extraction with acetonitrile containing 1% formic acid (6:1 solvent-to-sample ratio) and subsequent phospholipid removal using Ostro 96-well SPE plates (Waters). The workflow and HPLC-QTOF-MS acquisition parameters were identical to those applied to urine samples to ensure analytical comparability across matrices. Quality control was maintained through routine inclusion of blanks and pooled QC samples to monitor extraction reproducibility, instrument stability, and overall analytical performance.

Table S1. Urinary metabolites upregulated in adults with PKU.

M	Metabolite	Extracted mass	Assignation	RT (min)	FDR	VIP	Fragments MS/MS (*m/z*)	Biological source/pathway	MSI metabolite identification level
M01	Phenylalanine[a]	164.0698	$[M - H]^-$	1.02	2.28×10^{-18}	3.75	103.0556 $[M - H - CH_3NO_2]^-$, 77.0398 $[M - H - C_3H_5NO_2]^-$, 72.0088 $[M - H - C_7H_8]^-$	Phenylalanine metabolism	I
M02	2-Hydroxyphenylacetic acid	151.0393	$[M - H]^-$	2.98	4.57×10^{-18}	3.69	107.0495 $[M - H - CO_2]^-$, 64.0318[b] $[M - H - CO_2 - COCH_3]^-$	Phenylalanine metabolism	II
M03	Hydroxyphenylacetylglutamine	279.0949	$[M - H]^-$	2.33	6.62×10^{-14}	3.47	145.0618 $[M - H - C_8H_6O_2]^-$, 133.0293 $[M - H - C_5H_{10}N_2O_3]^-$, 127.0511 $[M - H - C_8H_6O_2 - H_2O]^-$	Phenylalanine metabolism	II
M04	Hydroxyphenylacetic acid glucuronide	327.0716	$[M - H]^-$	2.62	4.63×10^{-11}	3.20	151.0395 [M - H - glucuronide]$^-$, 107.0500 [M - H - glucuronide - $CO_2]^-$	Phenylalanine metabolism	II
M05	γ-Glutamylphenylalanine	295.1291	$[M + H]^+$	2.20	1.85×10^{-14}	3.14	120.0808 $[M + H - CO - H_2O - C_5H_7NO_3]^+$, 84.0443 $[M + H - CO - H_2O - C_9H_{11}NO_2]^+$	Phenylalanine metabolism	II
M06	Carboxyethylphenylalanine isomer 1	238.1074	$[M + H]^+$	1.98	5.50×10^{-14}	3.09	192.1018 $[M + H - CO - H_2O]^+$, 146.0963 $[M + H - 2\ CO - 2\ H_2O]^+$	Phenylalanine metabolism	II
M07	Carboxyethylphenylalanine isomer 2	238.1070	$[M + H]^+$	1.70	6.80×10^{-13}	3.07	192.1019 $[M + H - CO - H_2O]^+$, 146.0960 $[M + H - 2\ CO - 2\ H_2O]^+$	Phenylalanine metabolism	II
M08	N-lactoylphenylalanine[a]	238.1066	$[M + H]^+$	4.02	9.32×10^{-13}	3.02	120.0808 $[M + H - COOH - C_3H_5O_2]^+$, 103.0540 $[M + H - COOH - C_3H_5O_2 - NH_3]^+$	Phenylalanine metabolism	II
M09	Phenyllactic acid[a]	119.0490[c]	$[M - H - CO - H_2O]^-$	3.39	3.70×10^{-9}	3.01	117.0346 $[M - H - CH_4O_2]^-$, 101.0395 $[M - H - CH_4O_3]^-$, 72.9931 $[M - H - C_7H_8]^-$	Phenylalanine metabolism	II
M10	N-phenylacetylphenylalanine	282.1125	$[M - H]^-$	5.23	8.76×10^{-9}	3.00	164.0718 $[M - H - CO - C_7H_6]^-$, 147.0448 $[M - H - CO - C_7H_6$ -	Phenylalanine metabolism	II

							$NH_3]^-$, 103.0550 [M - H - CO - C_7H_6 - NH_3 - $CO_2]^-$, 72.0091 [M - H - CO - 2 $C_7H_7]^-$		
M11	N-acetylphenylalanine[a]	208.0971	$[M + H]^+$	3.64	1.07×10^{-12}	2.97	120.0813 [M + H - COOH - $COCH_3]^+$, 103.0547 [M + H - COOH - $COCH_3$ - $NH_3]^+$	Phenylalanine metabolism	II
M12	N-(ethoxyacetyl)phenylalanine isomer 1	250.1070	$[M - H]^-$	4.00	3.20×10^{-8}	2.93	164.0713 [M - H - $C_4H_6O_2$ $]^-$, 147.0445 [M - H - $C_4H_6O_2$ - $NH_3]^-$, 58.0299 [M - H - $C_{11}H_{12}O_3]^-$	Phenylalanine metabolism	II
M13	N-(ethoxyacetyl)phenylalanine isomer 2[a]	252.1231	$[M + H]^+$	3.65	7.92×10^{-10}	2.73	166.0858 [M + H - $C_4H_6O_2]^+$, 120.0805 [M + H - $C_4H_6O_2$ - CO - $H_2O]^+$	Phenylalanine metabolism	II
M14	Hydroxyphenylacetic acid sulfate[a]	230.9963	$[M - H]^-$	3.17	2.76×10^{-5}	2.36	151.0399 [M - H - $SO_3]^-$, 107.0505 [M - H - SO_3 - $CO_2]^-$, 96.9599 [M - H - $C_8H_6O_2]^-$	Phenylalanine metabolism	II
M15	N-phenylacetylglutamic acid[a]	266.1018	$[M + H]^+$	3.33	3.48×10^{-5}	2.02	130.0499 [M + H - C_8H_6O - $H_2O]^+$, 91.0544 [M + H - $C_6H_9NO_5]^+$, 84.0440 [M + H - C_8H_6O - 2 H_2O - $CO]^+$	Phenylalanine metabolism	II
M16	Phe-hexose[a]	328.1400	$[M + H]^+$	1.17	0.001	1.64	310.1286 [M + H - $H_2O]^+$, 292.1179 [M + H - 2 $H_2O]^+$, 264.1234 [M + H - 2 H_2O - $CO]^+$, 166.0865 [M + H - $C_6H_{10}O_5]^+$, 132.0812 [M + H - $C_5H_{10}O_5$ - CO - $H_2O]^+$, 120.0815 [M + H - $C_6H_{10}O_5$ - CO - $H_2O]^+$	Phenylalanine metabolism	II
M17	Phenylacetylglutamine[a]	265.1178	$[M + H]^+$	2.65	0.015	1.35	130.0499 [M + H - $C_8H_9NO]^+$, 91.0540 [M + H - $C_6H_{10}N_2O_4]^+$, 84.0441 [M + H - C_8H_9NO - CO - $H_2O]^+$	Phenylalanine metabolism	II
M18	8-Hydroxy-7-methylguanine	182.0687	$[M + H]^+$	0.74	7.90×10^{-15}	3.25	154.0725 [M + H - $CO]^+$, 126.0774 [M + H - 2 $CO]^+$	Purine metabolism	II

M19	Isoxanthopterin	180.0521	$[M + H]^+$	0.82	3.62×10^{-11}	2.97	163.0248 $[M + H - NH_3]^+$, 135.0300 $[M + H - NH_3 - CO]^+$, 110.0345 $[M + H - C_2H_2N_2O]^+$	Pteridine pathway	II
M20	Dihydrobiopterin	240.1073	$[M + H]^+$	0.67	7.82×10^{-5}	2.04	196.0845 $[M + H - C_2H_4O]^+$, 179.0795 $[M + H - C_2H_5O_2]^+$, 168.0877 $[M + H - C_2H_4O - CO]^+$, 165.0642 $[M + H - C_3H_7O_2]^+$	Pteridine pathway	II
M21	1H-Indole-3-carboxaldehyde	146.0594	$[M + H]^+$	4.08	7.25×10^{-12}	2.96	118.0657 $[M + H - CO]^+$, 91.0548 $[M + H - CO - HCN]^+$	Tryptophan metabolism	II
M22	Indolelactic acid[a]	206.0798	$[M + H]^+$	3.88	8.33×10^{-10}	2.75	130.0651 $[M + H - C_2H_4O_3]^+$, 118.0650 $[M + H - C_3H_4O_3]^+$	Tryptophan metabolism	I
M23	Indoleacetic acid	176.0708	$[M + H]^+$	4.54	1.21×10^{-6}	2.42	130.0658 $[M + H - CO - H_2O]^+$, 103.0546 $[M + H - CO - H_2O - HCN]^+$, 77.0390 $[M + H - C_4H_5NO_2]^+$	Tryptophan metabolism	I
M24	Phenylacetylcarnitine	280.1539	$[M + H]^+$	2.04	2.05×10^{-6}	2.30	85.0282 $[M + H - (CH_3)_3N - C_8H_8O_2]^+$	Acylcarnitine pathway	II
M25	4-Pyridoxic acid[a]	184.0604	$[M + H]^+$	0.99	2.17×10^{-5}	2.10	166.0492 $[M + H - H_2O]^+$, 148.0388 $[M + H - 2 H_2O]^+$	Vitamin B6 metabolism	II
M26	α-CEHC glucuronide	453.1742	$[M - H]^-$	5.07	0.001	2.04	277.1443 $[M - H - glucuronide]^-$, 233.1543 $[M - H - glucuronide - CO_2]^-$, 193.0350 $[M - H - C_{16}H_{20}O_3]^-$, 113.0241 $[M - H - glucuronide - H_2O - C_{11}H_{14}]^-$	Vitamin E metabolism	II
M27	α-CEHC	279.1594	$[M + H]^+$	4.43	1.38×10^{-4}	1.93	165.0908 $[M + H - C_6H_{10}O_2]^+$, 137.0952 $[M + H - C_7H_{10}O_3]^+$	Vitamin E metabolism	II
M28	Pantothenic acid	220.1170	$[M + H]^+$	1.47	0.004	1.71	124.0752 $[M + H - C_2H_8O_4]^+$, 98.0232 $[M + H - H_2O - C_5H_{12}O_2]^+$, 90.0546 $[M + H - C_6H_{10}O_3]^+$	Pantothenate biosynthesis	II
M29	1-Pyrroline-5-carboxylic acid	114.0568	$[M + H]^+$	0.59	0.024	1.39	96.0450 $[M + H - H_2O]^+$, 68.0499 $[M + H - CO - H_2O]^+$	Glutamate metabolism	II

Metabolites are ordered by VIP values within biological source/pathway. [a] FDR and VIP values refer to the most significant feature (i.e. fragment) of the compound (the lowest FDR). See Table S3. [b] Fragments were obtained by MS3 experiment. [c] It has been identified using autoMSMS and clustering. CEHC: carboxyethyl hydroxychroman, FDR: false discovery rate, MSI: Metabolomics Standards Initiative, Phe: phenylalanine, RT: retention time, VIP: variable importance in projection.

Table S2. Urinary metabolites downregulated in adults with PKU.

M	Metabolite	Extracted mass	Assignation	RT (min)	FDR	VIP	Fragments MS/MS (*m/z*)	Biological source/pathway	MSI metabolite identification level
M30	N-acetyl(iso)leucine	172.0963	$[M - H]^-$	2.97	5.51×10^{-13}	3.40	130.0871 $[M - H - COCH_2]^-$	Leucine, isoleucine and valine metabolism	II
M31	γ-Glutamyl(iso)leucine[a]	261.1432	$[M + H]^+$	1.76	0.012	1.44	132.1012 $[M + H - C_5H_7NO_3]^+$, 86.0962 $[M + H - C_5H_7NO_3 - CO - H_2O]^+$, 84.0439 $[M + H - H_2O - C_7H_{13}NO_3]^+$	Leucine, isoleucine and valine metabolism	II
M32	N-lactoyl(iso)leucine	202.1079	$[M - H]^-$	3.54	0.046	1.22	158.1181 $[M - H - CO_2]^-$, 130.0877 $[M - H - C_3H_4O_2]^-$	Leucine, isoleucine and valine metabolism	II
M33	Heptenoylcarnitine isomer 1	272.1862	$[M + H]^+$	2.60	1.25×10^{-9}	2.77	85.0286 $[M + H - (CH_3)_3N - C_7H_{12}O_2]^+$	Acylcarnitine pathway	II
M34	Hydroxyundecanoylcarnitine isomer	346.2589	$[M + H]^+$	4.81	3.54×10^{-7}	2.57	85.0285 $[M + H - (CH_3)_3N - C_{11}H_{22}O_3]^+$	Acylcarnitine pathway	II
M35	Octanoylcarnitine or methylheptanoylcarnitine or valproylcarnitine	288.2170	$[M + H]^+$	4.10	3.23×10^{-7}	2.47	85.0287 $[M + H - (CH_3)_3N - C_8H_{16}O_2]^+$	Acylcarnitine pathway	II
M36	Undecanoylcarnitine or dimethylnonanoylcarnitine or methyldecanoylcarnitine	330.2597	$[M + H]^+$	4.96	4.19×10^{-6}	2.36	85.0283 $[M + H - (CH_3)_3N - C_{11}H_{22}O_2]^+$	Acylcarnitine pathway	II
M37	Heptenoylcarnitine isomer 2	272.1851	$[M + H]^+$	3.15	5.96×10^{-6}	2.36	85.0280 $[M + H - (CH_3)_3N - C_7H_{12}O_2]^+$	Acylcarnitine pathway	II

M38	Octanoylcarnitine or methylheptanoylcarnitine or valproylcarnitine	288.2151	$[M + H]^+$	4.38	1.14×10^{-5}	2.24	85.0282 $[M + H - (CH_3)_3N - C_8H_{16}O_2]^+$	Acylcarnitine pathway	II
M39	Heptanoylcarnitine or methylhexanoylcarnitine	274.1995	$[M + H]^+$	3.41	3.18×10^{-5}	2.20	85.0281 $[M + H - (CH_3)_3N - C_7H_{14}O_2]^+$	Acylcarnitine pathway	II
M40	Decanoylcarnitine or methylnonanoylcarnitine	316.2467	$[M + H]^+$	4.89	2.91×10^{-5}	2.17	85.0287 $[M + H - (CH_3)_3N - C_{10}H_{20}O_2]^+$	Acylcarnitine pathway	II
M41	Dodecenoylcarnitine	342.2625	$[M + H]^+$	5.01	4.78×10^{-4}	1.93	85.0280 $[M + H - (CH_3)_3N - C_{12}H_{22}O_2]^+$	Acylcarnitine pathway	II
M42	Oxononanoylcarnitine or hydroxynonenoylcarnitine isomers	316.2108	$[M + H]^+$	1.86	2.23×10^{-4}	1.92	85.0283 $[M + H - (CH_3)_3N - C_9H_{16}O_3]^+$	Acylcarnitine pathway	II
M43	Oxononanoylcarnitine or hydroxynonenoylcarnitine isomers	316.2102	$[M + H]^+$	2.77	5.85×10^{-4}	1.90	85.0280 $[M + H - (CH_3)_3N - C_9H_{16}O_3]^+$	Acylcarnitine pathway	II
M44	Decanoylcarnitine or methylnonanoylcarnitine	316.2485	$[M + H]^+$	4.93	0.013	1.49	85.0284 $[M + H - (CH_3)_3N - C_{10}H_{20}O_2]^+$	Acylcarnitine pathway	II
M45	Nonenedioylcarnitine isomer	330.1916	$[M + H]^+$	2.30	0.016	1.44	85.0282 $[M + H - (CH_3)_3N - C_9H_{14}O_4]^+$	Acylcarnitine pathway	II
M46	N,N,N-trimethyltryptophan betaine	247.1448	$[M + H]^+$	2.16	9.77×10^{-9}	2.71	146.0599 $[M + H - (CH_3)_3N - COCH_2]^+$, 118.0652 $[M + H - C_6H_{11}NO_2]^+$, 60.0809 $[M + H - C_{11}H_9NO_2]^+$	Tryptophan metabolism	II
M47	Tryptophan[a]	205.0974	$[M + H]^+$	1.55	1.17×10^{-4}	1.99	188.0703 $[M + H - NH_3]^+$,146.0602 $[M + H - NH_3 - C_2H_2O]^+$, 118.0651 $[M + H - C_3H_5NO_2]^+$	Tryptophan metabolism	I
M48	Kynurenine[a]	209.0916	$[M + H]^+$	0.95	1.83×10^{-4}	1.98	146.0596 $[M + H - CH_5NO_2]^+$, 94.0647 $[M + H - C_4H_5NO_3]^+$	Tryptophan metabolism	I
M49	C-Glycosyltryptophan	367.1495	$[M + H]^+$	0.99	7.35×10^{-4}	1.81	332.1124 $[M + H - NH_3 - H_2O]^+$, 247.1067 $[M + H - C_4H_8O_4]^+$, 230.0811 $[M + H - C_4H_8O_4 -$	Tryptophan metabolism	II

							$NH_3]^+$, 202.0859 [M + H - $C_5H_8O_5$ - $NH_3]^+$, 188.0700 [M + H - $C_6H_{10}O_5$ - $NH_3]^+$		
M50	Indoleacetyl glutamine[a]	304.1303	$[M + H]^+$	3.14	0.004	1.65	130.0649 [M + H - $C_6H_{10}N_2O_4]^+$	Tryptophan metabolism	II
M51	Kynurenic acid[a]	190.0509	$[M + H]^+$	2.76	0.042	1.18	144.0439 [M + H - CO - $H_2O]^+$, 116.0493 [M + H - 2 CO - $H_2O]^+$, 89.0384 [M + H - $C_3H_3NO_3]^+$	Tryptophan metabolism	I
M52	5-Hydroxyindoleacetic acid	192.0665	$[M + H]^+$	2.34	0.038	1.12	146.0600 [M + H - CO - $H_2O]^+$, 118.0656 [M + H - 2 CO - $H_2O]^+$, 91.0547 [M + H - $C_3H_3NO_3]^+$	Tryptophan metabolism	II
M53	1-Methylhistidine	170.0921	$[M + H]^+$	0.27	9.61×10^{-8}	2.64	124.0865 [M + H - CO - $H_2O]^+$, 109.0756 [M + H - $CH_3NO_2]^+$, 96.0683 [M + H - $C_2H_4NO_2]^+$	Animal protein consumption	I
M54	5-Aminovaleric acid betaine	160.1320	$[M + H]^+$	0.32	6.21×10^{-7}	2.35	101.0598 [M + H - $(CH_3)_3N]^+$, 60.0810 [M + H - $C_5H_8O_2]^+$, 55.0544 [M + H - $(CH_3)_3N$ - CO - $H_2O]^+$	Animal protein consumption	II
M55	3-Methylhistidine	170.0915	$[M + H]^+$	0.22	3.39×10^{-6}	2.30	109.0753 [M + H - $CH_3NO_2]^+$, 96.0862 [M + H - $C_2H_4NO_2]^+$	Animal protein consumption	I
M56	Creatine	132.0765	$[M + H]^+$	0.31	0.017	1.26	90.0545 [M + H - $CH_2N_2]^+$	Animal protein consumption	I
M57	Dihydroxybenzoic acid isomer	153.0184	$[M - H]^-$	4.91	7.05×10^{-6}	2.60	135.0091 [M - H - $H_2O]^-$, 109.0298 [M - H - $CO_2]^-$, 91.0188 [M - H - CO_2 -$H_2O]^-$, 67.0196 [M - H - $C_3H_2O_3]^-$, 65.0401 [M - H - 2 $CO_2]^-$	Drug - topical agent	II
M58	Urolithin B glucuronide	387.0720	$[M - H]^-$	4.78	3.41×10^{-5}	2.48	211.0401 [M - H - glucuronide]$^-$, 167.0499[b] [M - H - glucuronide - $CO_2]^-$, 139.0547[b] [M - H - glucuronide - CO_2 - CO]$^-$	Phenolic compound	II

M59	Dihydroxy-H-indole glucuronide isomer 1	324.0718	$[M - H]^-$	1.95	8.49×10^{-4}	2.09	193.0354 $[M - H - C_8H_5NO]^-$, 160.0406 $[M - H - C_5H_8O_6]^-$, 148.0402 [M - H - glucuronide]$^-$, 113.0244 $[M - H - C_6H_{11}O_8]^-$, 72.9933 $[M - H - C_{12}H_{13}NO_5]^-$, 59.0144 $[M - H - C_{12}H_{11}NO_6]^-$	Phenolic compound	II
M60	Enterolactone glucuronide	473.1452	$[M - H]^-$	4.89	0.001	2.03	297.1136 [M - H - glucuronide]$^-$, 253.1238 [M - H - glucuronide - $CO_2]^-$	Phenolic compound	II
M61	Urolithin A glucuronide	403.0649	$[M - H]^-$	3.84	8.07×10^{-4}	1.96	227.0351 [M - H - glucuronide]$^-$, 198.0314[b] $[M - H - C_7H_9O_7]^-$, 182.0366[b] $[M - H - C_7H_9O_8]^-$, 171.0439[b] $[M - H - C_8H_8O_8]^-$, 159.0443[b] $[M - H - C_9H_8O_8]^-$	Phenolic compound	II
M62	Dihydroxy-H-indole glucuronide isomer 2	324.0718	$[M - H]^-$	2.06	0.001	1.96	193.0352 $[M - H - C_8H_5NO]^-$, 160.0406 $[M - H - C_5H_8O_6]^-$, 148.0401 [M - H - glucuronide]$^-$, 113.0244 $[M - H - C_6H_{11}O_8]^-$, 72.9932 $[M - H - C_{12}H_{13}NO_5]^-$, 59.0142 $[M - H - C_{12}H_{11}NO_6]^-$	Phenolic compound	II
M63	Hepteneoylglycine isomer	184.098	$[M - H]^-$	3.36	7.00×10^{-5}	2.33	74.0248 $[M - H - C_7H_{10}O]^-$	Glycine compound	II
M64	Methylbutyrylglycine or isovalerylglycine or valerylglycine	158.0805	$[M - H]^-$	1.95	0.013	1.72	74.0247 $[M - H - C_5H_8O]^-$	Glycine compound	II
M65	Pyrraline[a]	255.1324	$[M + H]^+$	1.51	1.77×10^{-5}	2.09	175.1226 $[M + H - CH_4O_4]^+$, 148.1119 $[M + H - C_2H_5NO_4]^+$	Food component	II
M66	N2,N5-Diacetylornithine[a]	215.1028	$[M - H]^-$	0.98	0.012	1.71	173.0932 $[M - H - COCH_2]^-$, 131.0826 $[M - H - 2\ COCH_2]^-$, 58.0301 $[M - H - C_7H_{11}NO_3]^-$	Urea cycle, arginine and proline metabolism	II

M67	1,7-Dimethyluric acid	195.0507	$[M - H]^-$	1.82	0.010	1.64	180.0286 $[M - H - CH_3]^-$, 137.0228 $[M - H - C_2H_4NO]^-$	Caffeine metabolism	II
M68	1,3,7-Trimethyluric acid	211.0821	$[M + H]^+$	2.15	0.003	1.53	196.0582 $[M + H - CH_3]^+$, 154.0606 $[M + H - C_2H_3NO]^+$, 126.0655 $[M + H - C_2H_3NO - CO]^+$	Caffeine metabolism	II
M69	Caffeine	195.0866	$[M + H]^+$	2.53	0.017	1.27	138.0657 $[M + H - C_2H_3NO]^+$, 110.0707 $[M + H - C_2H_3NO - CO]^+$, 83.0603 $[M + H - C_4H_4N_2O_2]^+$, 69.0448 $[M + H - C_5H_6N_2O_2]^+$	Caffeine metabolism	I
M70	Paraxanthine	181.0709	$[M + H]^+$	1.91	0.049	1.07	124.0499 $[M + H - C_2H_3NO]^+$, 69.0448 $[M + H - C_4H_4N_2O_2]^+$, 55.0291 $[M + H - C_5H_6N_2O_2]^+$	Caffeine metabolism	II
M71	N-acetylaspartylglutamic acid[a]	305.0952	$[M + H]^+$	0.79	0.005	1.54	148.0600 $[M + H - C_6H_7NO_4]^+$, 130.0495 $[M + H - C_6H_9NO_5]^+$, 102.0545 $[M + H - C_6H_7NO_4 - CO - H_2O]^+$, 88.0391 $[M + H - C_6H_7NO_4 - C_2H_4O_2]^+$, 84.0442 $[M + H - C_6H_9NO_5 - CO - H_2O]^+$	Alanine, aspartate and glutamate metabolism	II
M72	Hexanoylglutamine[a]	245.1506	$[M + H]^+$	3.19	0.006	1.51	130.0498 $[M + H - C_6H_{13}NO]^+$, 84.0442 $[M + H - C_6H_{13}NO - CO - H_2O]^+$	Glutamine metabolism	II
M73	Tyrosine[a]	182.0808	$[M + H]^+$	0.57	0.017	1.33	136.0760 $[M + H - CO - H_2O]^+$, 123.0430 $[M + H - NH_3 - C_2H_2O]^+$	Phenylalanine metabolism	I

Metabolites are ordered by VIP values within biological source/pathway. [a] FDR and VIP values refer to the most significant feature (i.e. fragment) of the compound (the lowest FDR). See Table S4. [b] Fragments were obtained by MS3 experiment. FDR: false discovery rate, MSI: Metabolomics Standards Initiative, RT: retention time, VIP: variable importance in projection.

Table S3. Significant mass features from Table S1.

M	Metabolite	Extracted mass [assignation]
M01	Phenylalanine	72.0086 [M - H - C_7H_8]$^-$
M08	N-lactoylphenylalanine	120.0805 [M + H - COOH - $C_3H_5O_2$]$^+$
M09	Phenyllactic acid	119.0490 [M - H -CO - H_2O]$^-$
M11	N-acetylphenylalanine	162.0911 [M + H - CO - H_2O]$^+$
M13	N-(ethoxyacetyl)phenylalanine isomer 2	234.1130 [M + H - H_2O]$^+$
M14	Hydroxyphenylacetic acid sulfate	151.0396 [M - H - SO_3]$^-$
M15	N-phenylacetylglutamic acid	148.0614 [M + H - C_8H_6O]$^+$
M16	Phe-hexose	310.1277 [M + H - H_2O]$^+$
M17	Phenylacetylglutamine	147.0763 [M + H - C_8H_6O]$^+$;
M20	Dihydrobiopterin	196.0855 [M + H - C_2H_4O]$^+$
M22	Indolelactic acid	160.0740 [M + H - CO - H_2O]$^+$
M25	4-Pyridoxic acid	166.0493 [M + H - H_2O]$^+$

Phe, phenylalanine.

Table S4. Significant mass features from Table S2.

M	Metabolite	Extracted mass [assignation]
M31	γ-Glutamyl(iso)leucine	132.1026 $[M + H - C_5H_7NO_3]^+$
M47	Tryptophan	118.0642 $[M + H - NH_3 - CH_2CO - CO]^+$
M48	Kynurenine	192.0667 $[M + H - NH_3]^+$
M50	Indoleacetyl glutamine	158.0611 $[M + H - C_5H_{10}N_2O_3]^+$
M51	Kynurenic acid	162.0537 $[M + H - CO]^+$
M65	Pyrraline	237.1234 $[M + H - H_2O]^+$
M66	N2,N5-Diacetylornithine	173.0904 $[M - H - COCH_2]^-$
M71	N-acetylaspartylglutamic acid	148.0598 $[M + H - C_6H_7NO_4]^+$
M72	Hexanoylglutamine	147.0771 $[M + H - C_6H_{10}O]^+$
M73	Tyrosine	136.0755 $[M + H - H_2O - CO]^+$

Table S5. Receiver operating characteristic (ROC) performance metrics for urinary metabolites.

M	Metabolite	AUC	Sensitivity	Specificity
M18	8-Hydroxy-7-methylguanine	0.98862	0.921053	0.972973
M01	Phenylalanine	0.97795	0.947368	1.0
M05	γ-Glutamylphenylalanine	0.97368	0.947368	0.945946
M02	2-Hydroxyphenylacetic acid	0.96728	0.947368	1.0
M06	Carboxyethylphenylalanine isomer 1	0.96373	0.894737	0.972973
M30	N-acetyl(iso)leucine	0.96373	0.947368	0.945946
M07	Carboxyethylphenylalanine isomer 2	0.95875	0.868421	0.945946
M08	N-lactoylphenylalanine	0.95377	0.868421	0.918919
M19	Isoxanthopterin	0.93883	0.894737	0.837838
M03	Hydroxyphenylacetylglutamine	0.92817	0.894737	0.891892
M11	N-acetylphenylalanine.	0.92603	0.894737	0.891892
M21	1H-Indole-3-carboxaldehyde	0.92603	0.868421	0.945946
M10	N-phenylacetylphenylalanine	0.92354	0.868421	0.972973
M04	Hydroxyphenylacetic acid glucuronide	0.9175	0.815789	0.918919
M33	Heptenoylcarnitine isomer 1	0.90612	0.894737	0.864865

AUC, area under the curve.

Table S6. HPLC-QTOF-MS parameters.

<table>
<tr><td colspan="4">Acquisition Mode</td></tr>
<tr><td colspan="2">Min Range (m/z)</td><td colspan="2">50</td></tr>
<tr><td colspan="2">Max Range (m/z)</td><td colspan="2">1200</td></tr>
<tr><td colspan="2">Scan Rate (spectra/sec)</td><td colspan="2">2.0</td></tr>
<tr><td colspan="4">Instrument Parameters</td></tr>
<tr><td colspan="2">Gas Temp (°C)</td><td colspan="2">300</td></tr>
<tr><td colspan="2">Gas Flow (l/min)</td><td colspan="2">5</td></tr>
<tr><td colspan="2">Nebulizer (psig)</td><td colspan="2">35</td></tr>
<tr><td colspan="2">SheathGasTemp</td><td colspan="2">350</td></tr>
<tr><td colspan="2">SheathGasFlow</td><td colspan="2">11</td></tr>
<tr><td colspan="4">Scan Source Parameters</td></tr>
<tr><td colspan="2">VCap</td><td colspan="2">3500</td></tr>
<tr><td colspan="2">Nozzle Voltage (V)</td><td colspan="2">1000</td></tr>
<tr><td colspan="2">Fragmentor</td><td colspan="2">400</td></tr>
<tr><td colspan="2">Skimmer1</td><td colspan="2">65</td></tr>
<tr><td colspan="2">OctopoleRFPeak</td><td colspan="2">750</td></tr>
<tr><td colspan="4">Sampling speed</td></tr>
<tr><td colspan="2">Draw Speed (μL/min)</td><td colspan="2">100.0</td></tr>
<tr><td colspan="2">Eject Speed (μL/min)</td><td colspan="2">400.0</td></tr>
<tr><td colspan="2">Wait Time After Drawing (s)</td><td colspan="2">1.2</td></tr>
<tr><td colspan="4">Injection</td></tr>
<tr><td colspan="2">Injection Volume (μL)</td><td colspan="2">5.00</td></tr>
<tr><td colspan="4">Timetable</td></tr>
<tr><td>Time</td><td>A</td><td>B</td><td>Flow</td></tr>
<tr><td>4 min</td><td>80.00 %</td><td>20.00%</td><td>0.600 mL/min</td></tr>
<tr><td>6 min</td><td>5.00 %</td><td>95.00 %</td><td>0.600 mL/min</td></tr>
<tr><td>7.5 min</td><td>5.00 %</td><td>95.00 %</td><td>0.600 mL/min</td></tr>
<tr><td>8 min</td><td>99.00 %</td><td>1.00 %</td><td>0.600 mL/min</td></tr>
<tr><td>12 min</td><td>99.00 %</td><td>1.00 %</td><td>0.600 mL/min</td></tr>
</table>

Table S7. Agilent MassHunter Profinder 10.0.02 software parameters for batch feature extraction of urine samples.

Features were extracted using the Batch Recursive Feature Extraction module for small molecules (8 steps). A .CEF file was generated.

Parameter	ESI (+)	ESI (-)
Step 1 of 8: MFE – Extraction Parameters		
Retention time (RT)	0.0 – 5.5 min	0.0 – 5.5 min
Peaks with height	>= 5000 counts	>= 200 counts
Ion Species	+H	-H, -HCOO
Isotope model	Common organic (no halogens)	Common organic (no halogens)
Charge state	Limit assigned charge states to a range of 1-2	Limit assigned charge states to a range of 1-2
Step 2 of 8: MFE – Compound Filters		
Compound ion count threshold	Two or more ions	Two or more ions
Step 3 of 8: Compound Binning and Alignment		
RT tolerance	± (0.00% + 0.15 min)	± (0.00% + 0.10 min)
Mass tolerance	± (7.00 ppm + 2.50 mDa)	± (7.00 ppm + 2.50 mDa)
Step 4 of 8: MFE – Post-Processing Filters		
Absolute height	>= 10000 counts	>= 10000 counts
Score (MFE)	80.00	70.00
Minimum filter matches	A compound must satisfy the checked MFE filter conditions	A compound must satisfy the checked MFE filter conditions

	in at least 2 file(s) in at least one sample group	in at least 1 file(s) in at least one sample group
Global filters	Limit to the largest 5000 compound group(s)	Limit to the largest 5000 compound group(s)
Step 5 of 8: Find by Ion – Matching Tolerances and Scoring		
Match tolerance - Masses	± 10.00 ppm	± 2.50 mDa
Match tolerance - RT	± 0.15 min	± 0.15 min
Possible m/z	Symmetric (ppm) ± 35	Symmetric (m/z) ± 0.025
Limit EIC extraction range	Yes	Yes
Expected RT	Symmetric ± 1.50 min	Symmetric ± 1.50 min
Step 6 of 8: Find by Ion – EIC Peak Integration and Filtering		
Integration	Agile 2	Agile 2
Filter on	Peak height	Peak height
Chromatogram data format	Centroid when available, otherwise Profile	Centroid when available, otherwise Profile
Step 7 of 8: Find by Ion – Spectrum Extraction and Centroiding		
Spectra to include	Average scans > 10% of peak height	Average scans > 10% of peak height
TOF spectra	Exclude if above 20.0% of saturation	Exclude if above 20.0% of saturation
Maximum spike width	2	2
Required valley	0.70	0.70
Mass spectral data format	Centroid when available, otherwise Profile	Centroid when available, otherwise Profile
Step 8 of 8: Find by Ion – Post-Processing Filters		

Absolute height	>= 10000 counts	>= 10000 counts
Score (Tgt)	>= 50.00	>= 50.00
Minimum filter matches	A compound must satisfy the checked Find by Ion filter conditions in at least 2 file(s) in at least one sample group	A compound must satisfy the checked Find by Ion filter conditions in at least 1 file(s) in at least one sample group
Global filters	Limit to the largest 5000 compound group(s)	Limit to the largest 5000 compound group(s)

EIC, extracted ion chromatogram; MFE, molecular feature extraction; RT, retention time; TOF, time-of-flight; Tgt, score value composed of the score of MS and RT.

Table S8. Parameters for Agilent Mass Profiler Profesional (MPP) experiments of urine samples.

CEF files generated from Agilent Masshunter Profinder were used in MPP 15.1 software to create a raw .txt file to use it in MetaboAnalyst 6.0 for statistical analysis

Parameter	ESI (+)	ESI (-)
Minimum absolute abundance	5000 counts	5000 counts
Number of ions	Minimum number of ions 1	Minimum number of ions 1
Charge states	All charge states permitted	All charge states permitted
RT Window	0% + 0.15min	0% + 0.05min
Mass Window	0.05 ppm + 2mDa	0.15 ppm + 2mDa
Features from Profinder	4887	4984
Features from MPP	4887	4984

ESI, electrospray-ionization; RT, retention time.

Figure S1. PCA scores plot in 2D (PC1 vs PC2) of population urine samples in ESI+ (Figure 1A) and ESI- (Figure 1B). PC1: 13.8% and 8.5%, PC2: 6.9% and 6.7%, for ESI+ and ESI- respectively. CON: control group, PKU: PKU group.

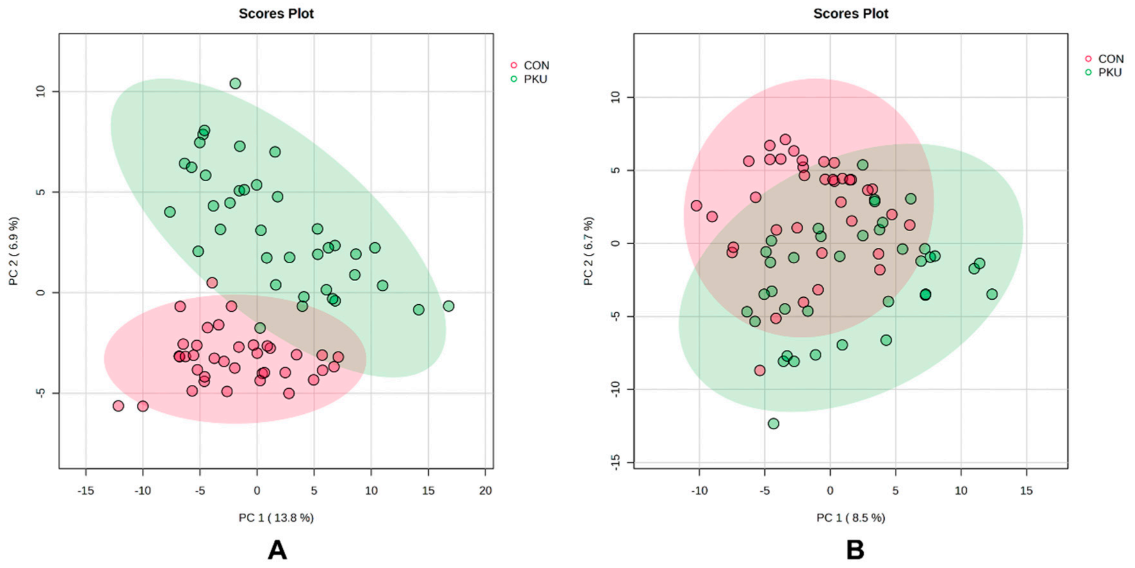

Figure S2. Permutation test of the OPLS-DA ESI+ (Figure A) and ESI- models (Figure B) of urine samples.

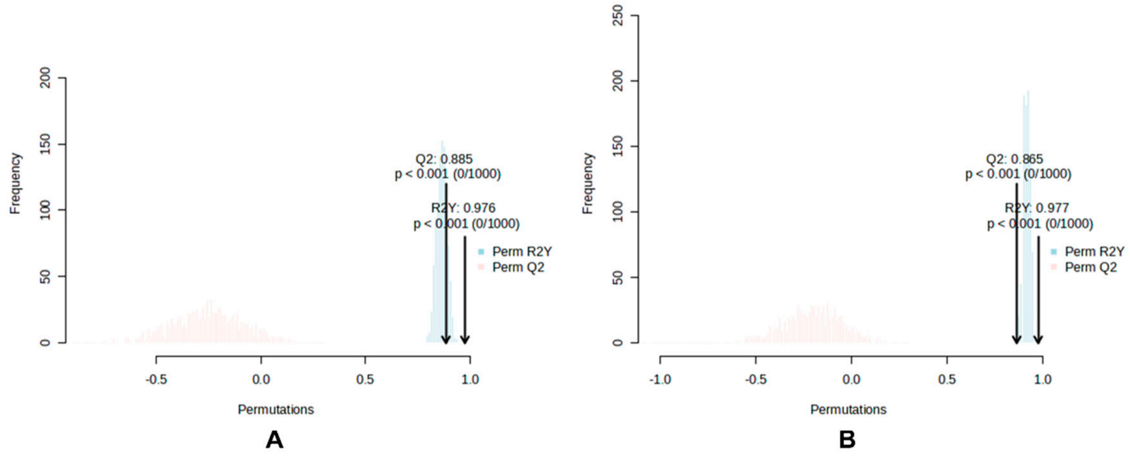

Figure S3. Quantitative Enrichment Analysis of differential metabolites using urinary metabolite sets of disease signature.

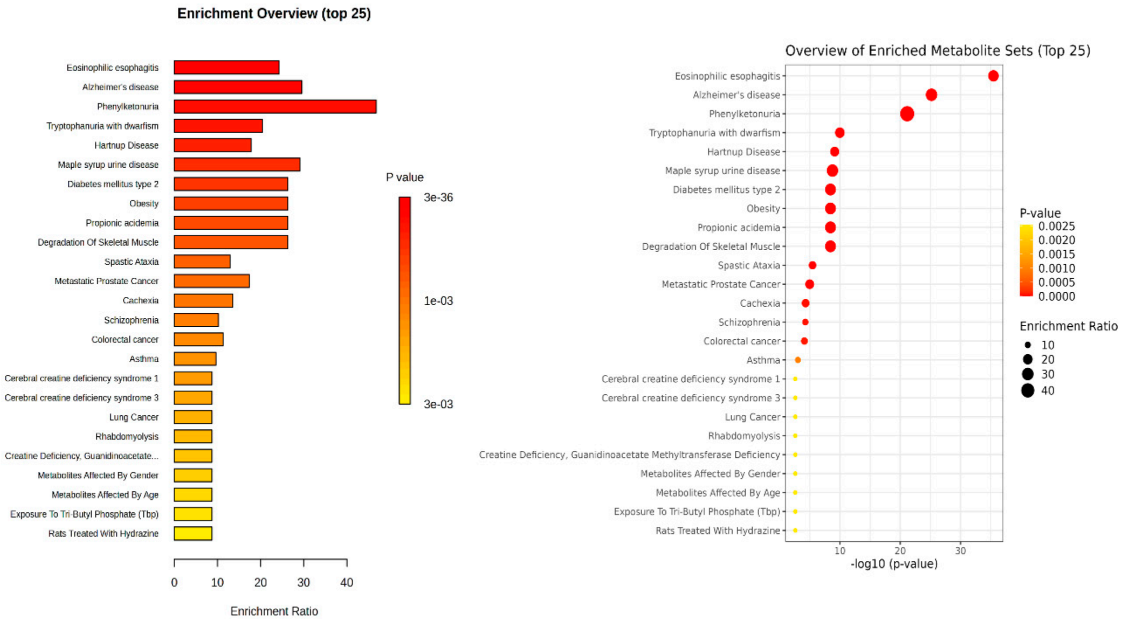

Metabolites detected in the urinary metabolite sets identified Phenylketonuria (PKU) as the disease with the highest enrichment ratio (46.8) and the third most significant pathway. Eosinophilic esophagitis and Alzheimer's disease showed higher FDR values than PKU (1.22 x 10^{-34} vs. 1.13 x 10^{-24} vs. 4.18 x 10^{-21}). Interestingly, other conditions, such as diabetes, obesity or skeletal muscle degradation, were also enriched in the control group, as 1- and 3-methylhistidine metabolites, which are increased in this population, were the only metabolites involved in these pathways.

References

1. Tulipani, S.; Llorach, R.; Urpi-Sarda, M.; Andres-Lacueva, C. Comparative Analysis of Sample Preparation Methods to Handle the Complexity of the Blood Fluid Metabolome: When Less Is More. Anal Chem **2013**, 85, 341–348, doi:10.1021/ac302919t.

2. Tulipani, S.; Mora-Cubillos, X.; Jáuregui, O.; Llorach, R.; García-Fuentes, E.; Tinahones, F.J.; Andres-Lacueva, C. New and Vintage Solutions to Enhance the Plasma Metabolome Coverage by LC-ESI-MS Untargeted Metabolomics: The Not-so-Simple Process of Method Performance Evaluation. Anal Chem **2015**, 87, 2639–2647, doi:10.1021/ac503031d.
